# Feasibility of enhancing well‐child visits with family nutrition and physical activity risk assessment on body mass index

**DOI:** 10.1002/osp4.339

**Published:** 2019-04-24

**Authors:** L. Bailey‐Davis, S. M. R. Kling, G. C. Wood, W. J. Cochran, J. W. Mowery, J. S. Savage, R. A. Stametz, G. J. Welk

**Affiliations:** ^1^ Geisinger Obesity Institute Geisinger Danville PA USA; ^2^ Department of Nutritional Sciences The Pennsylvania State University, University Park State College PA USA; ^3^ Gastroenterology Geisinger Danville PA USA; ^4^ Center for Childhood Obesity Research, Department of Nutritional Sciences The Pennsylvania State University, University Park State College PA USA; ^5^ Steele Institute for Health Innovation Geisinger Danville PA USA; ^6^ Department of Kinesiology Iowa State University Ames IA USA

**Keywords:** BMI, paediatrics, prevention, primary care

## Abstract

**Objective:**

Integration of behavioural risk assessment into well‐child visits is recommended by clinical guidelines, but its feasibility and impact is unknown.

**Methods:**

A quasi‐experimental study evaluated the feasibility and effectiveness of risk assessment on body mass index (BMI) at 1‐year follow‐up. Children with assessments (intervention) were compared with those who did not complete assessments (non‐respondent) and those who received standard care (non‐exposed).

**Results:**

Analyses included 10,647 children aged 2–9 years (2,724 intervention, 3,324 non‐respondent and 4,599 non‐exposed). Forty‐five per cent of parents completed the assessments. Intervention and non‐respondent groups differed in change in BMI *z*‐score at 1 year by −0.05 (confidence interval [CI]: −0.08, −0.02; *P* = 0.0013); no difference was observed with non‐exposed children. The intervention group had a smaller increase in BMI *z*‐score (0.07 ± 0.63) than non‐respondent group (0.13 ± 0.63). For children with normal weight at baseline, intervention versus non‐respondent groups differed in BMI *z*‐score change by −0.06 (CI: −0.10, −0.02; *P* = 0.0025). However, children with overweight at baseline in the intervention versus the non‐exposed group differed in BMI *z*‐score change (0.07 [CI: 0.02, 0.14]; *P* = 0.016). When analysed by age, results were similar for 2‐ to 5‐year‐olds, but no differences were found for 6‐ to 9‐year‐olds.

**Conclusion:**

Automating risk assessment in paediatric care is feasible and effective in promoting healthy weight among preschool but not older children.

## Introduction

Childhood obesity remains a pervasive problem, and in response, obesity prevention guidance has been established for paediatric primary care 1. Endorsed universal protocols for identification, prevention and treatment of paediatric obesity include body mass index (BMI) screening, risk assessment, preventive counselling and education for parents and children, regardless of weight status 2, 3. Screening should include growth (e.g. age‐specific and sex‐specific BMI percentile) and risk assessment of factors including parental weight status, family income, nutrition, physical and sedentary activity, and sleep 3.

Paediatric primary care providers can play a critical role by improving parental understanding of healthy growth patterns and obesity risk factors 4. Mothers prefer physicians as the source of feeding, growth and health information, and paediatric providers agree it is their role to discuss these topics 5, 6, 7. Primary prevention strategies to minimize risk may be more effective and efficient than treating obesity after it occurs. Because most children attend well‐child visits (WCVs) and paediatric providers are trusted healthcare professionals 4, the paediatric clinical setting provides a promising, broad‐scale opportunity to conduct primary obesity prevention. To capitalize on this opportunity, it is important to develop and test tools that can be integrated into the providers' practice and workflow 8.

The family nutrition and physical activity (FNPA) risk assessment tool could serve as a standardized tool to meet these clinical guidelines as it assesses parenting practices, child behaviours and home environmental characteristics that predispose children to becoming obese 9. In school‐aged children, the tool demonstrates utility in longitudinal analyses to predict a child's risk for obesity 10, 11, and the summary risk score has been related to adiposity measures, severity of obesity, cardiovascular disease risk and glucose intolerance 12, 13, 14. Furthermore, the FNPA has been shown to enable paediatric providers to quickly assess risk and provide behaviourally anchored counselling during WCVs 15.

The present study examined the feasibility of collecting FNPA assessments under real‐world conditions and the effectiveness of integrating the assessment during WCV on child BMI over a 1‐year period. Given that exposure to FNPA has been associated with parent intent to reduce risk behaviours associated with childhood obesity 16, the hypothesis was that children whose parents completed the FNPA at baseline would have lower BMI at follow‐up when compared with children whose parents did not complete FNPA, regardless of whether this was due to failure or lack of opportunity to respond. Results are reported by child baseline weight category to evaluate the effectiveness of the intervention on primary and secondary prevention. Additionally, we report associations between FNPA risk assessment score and obesity risk to validate FNPA in a new population, preschool‐aged children 9, 11. Likewise, results for preschool‐aged and school‐aged children are reported separately given that FNPA was originally designed for school‐aged children.

## Methods

### Experimental design

A quasi‐experimental study design was used to evaluate the effectiveness of the multilevel intervention that consisted of (a) health system programming that fired the FNPA risk assessment at scheduled WCVs, captured parent‐reported data and integrated those data into the child's electronic health record (EHR) as clinical decision support; (b) parent completion of the risk assessment; and (c) provider use of the child's EHR to observe the risk assessment data and deliver preventive counselling. The feasibility of collecting FNPA data at WCV was evaluated as the frequency of parents being offered and completing the assessment in association with a scheduled WCV. Change in BMI *z*‐score at 1‐year follow‐up was the primary outcome.

### Participants

Intervention group participants included children who had a baseline WCV at an intervention clinic between 1 November 2013 and 31 October 2014 with weight and height data (BMI screening) and a completed FNPA assessment and a second (1‐year follow‐up) WCV with height and weight data within 10–18 months of baseline. The non‐respondent group participants were children from the same clinics as intervention participants and FNPA assessments were offered but not completed at baseline. Non‐exposed group participants included children who completed a baseline WCV at a non‐intervention clinic with weight and height data between 1 November 2013 and 31 October 2014 and had follow‐up WCVs within the described timeline earlier. Participants were aged 2–9 years at baseline. Child sex, date of birth, height, weight, FNPA completion status, demographic information and clinical diagnoses were extracted from the EHR. Children diagnosed with type 1 diabetes or cancer were excluded.

The participants were derived from paediatric clinics at Geisinger, a large, integrated health system in Pennsylvania. The health system made the FNPA risk assessment available as standard of care during WCVs in conveniently selected intervention clinics, and all providers in those clinics had been trained to use the FNPA. Intervention and non‐respondent participants were derived from 14 clinics, and all providers were employed by Geisinger. Non‐exposed participants were derived from six clinics where FNPA was not available nor were providers trained on the intervention. The intervention group was compared with both control groups separately.

### Risk assessment intervention

The intervention was designed to promote assessment of behavioural and environmental risk factors for obesity and to help parents and providers engage in discussion about healthy lifestyles. The intervention was initiated with an automated email generated 10 days prior to a scheduled WCV via the patient portal (at home). The email included a link to the FNPA risk assessment. As shown in Table 1, the 20‐item tool includes 10 constructs predictive of childhood obesity 9, 10, 11.

**Table 1:** Family nutrition and physical activity risk assessment tool

For each question, please select the answer that best represents your child/family				
	Almost never	Sometimes	Usually	Almost always
1. My child eats breakfast …	□	□	□	□
2. Our family eats meals together …	□	□	□	□
3. Our family eats while watching TV …	□	□	□	□
4. Our family eats fast food …	□	□	□	□
5. Our family uses microwave or ready to eat foods …	□	□	□	□
6. My child eats fruits and vegetables at meals or snacks …	□	□	□	□
7. My child drinks soda pop or sugar drinks …	□	□	□	□
8. My child drinks low fat milk at meals or snacks …	□	□	□	□
9. Our family limits eating of chips, cookies, and candy …	□	□	□	□
10. Our family uses candy as a reward for good behavior …	□	□	□	□
11. My child spends less than 2 hours on TV/games/computer per day …	□	□	□	□
12. Our family limits the amount of TV our child watches …	□	□	□	□
13. Our family allows our child to watch TV in their bedroom …	□	□	□	□
14. Our family provides opportunities for physical activity	□	□	□	□
15. Our family encourages our child to be active every day	□	□	□	□
16. Our family finds ways to be physically active together …	□	□	□	□
17. My child does physical activity during his/her free time …	□	□	□	□
18. My child is enrolled in sports or activities with a coach or leader …	□	□	□	□
19. Our family has a daily routine for our child's bedtime …	□	□	□	□
20. My child gets 9 hours of sleep a night …	□	□	□	□

Parents completed the FNPA risk assessment in the patient portal or waiting room (via iPad, kiosk) and received immediate feedback that displayed their response, the recommended practice and a behaviourally anchored strategy to consider adopting. At home, parents could print results; in the clinic, this was incorporated into the printed summary report provided after the visit. The risk assessment took less than 2 min to complete, a process acceptable to both parents and providers 15. Front desk support staff and rooming nurses at intervention clinics were trained to encourage parent completion of the risk assessment before the patient was roomed for examination.

The health system's programming enabled automated FNPA risk assessment data collection; integration of these data with BMI screening data into the EHR in real‐time; and display as clinical decision support. This support included responses for each FNPA item, the summary score, talking points for promoting health and reducing risk, and printable educational materials. Providers were trained to use motivational interviewing and goal setting to counsel parents on the FNPA topics and provide tailored feedback with educational materials. Educational materials (English and Spanish) were mapped to FNPA topics and developed by the Academy of Nutrition and Dietetics Foundation Kids Eat Right Program 17.

### Anthropometric measures

All clinics were trained on anthropometric methods for height and weight measurement and used standardized, calibrated scales (Healthometer 599KL) and stadiometers (SECA 264) to screen BMI with EHR documentation. As a standard procedure at WCVs, height was measured to the nearest 0.1 cm and weight to the nearest 0.1 kg. Sex‐specific BMI‐for‐age percentiles were calculated in EHR to identify the children by weight status: underweight (≤5th percentile), normal weight (>5th and <85th), overweight (≥85th and <95th), obese (≥95th and <99th) and severely obese (≥99th).

### Statistical analysis

All analyses were performed using a sas (version 9.4) statistical software (SAS Institute, Inc., Cary, NC). Differences in the baseline demographic characteristics between the intervention, non‐respondent and non‐exposed groups were assessed. Categorical variables (e.g. race) were compared using chi‐squared test, and continuous variables (e.g. age and BMI *z*‐score) were tested for normality. If variables were normally distributed, then *t*‐tests were used. Mann–Whitney *U*‐tests were used to test for nonparametric differences in distribution.

Outcomes of interest were frequency of FNPA risk assessment completion by opportunity, odds of obesity by FNPA risk score tertile and changes in BMI *z*‐score from baseline to 1‐year follow‐up WCV. An analysis of variance was used to determine the association between weight status category and mean baseline FNPA risk score. Logistic regression was used to calculate the odds of obesity by baseline FNPA risk score tertile to evaluate validity of the tool. Differences in BMI *z*‐score at baseline and 1 year were compared between groups using two‐sample *t*‐tests. Generalized linear regression models were used to test for differences in change in BMI *z*‐score between groups. *Post hoc* analyses used multilevel models to examine and account for clustering within clinic site and care provider. For these models, the intraclass correlation coefficient was calculated to evaluate the amount of variation explained by site and provider clusters. This analysis included the introduction of clinic‐level characteristics (clinic‐level tertiles for the number of WCVs per year, per cent of WCV with completed FNPA, number of providers and number of WCV per provider) and provider characteristic (tertile for number of WCVs per year) to test for site‐level and provider‐level effects and effect modification with the intervention. Data are reported as means ± standard deviations, and results were considered significant at *P* < 0.05.

## Results

#### Participant characteristics

As shown in Table 2a, 2,724 intervention, 3,324 non‐respondent and 4,599 non‐exposed children were evaluated. Children were primarily non‐Hispanic White and male, 27.2% received medical assistance (a proxy for low‐income status) and 12.9% of children were obese, which is lower than national estimates 1. Demographics were similar for the two age groups of 2–5 years (Table 2b) and 6–9 years (Table 2c) at baseline. As shown, a total of 6,496 children were aged 2–5 (1,617 intervention, 2,133 non‐respondent and 2,746 non‐exposed), and 4,151 were aged 6–9 (1,107 intervention, 1,191 non‐respondent and 1,853 non‐exposed). At baseline, on average, the intervention children significantly differed from the non‐respondent and the non‐exposed children in age, BMI *z*‐score and weight category (all *P*'s ≤ 0.05). Intervention and non‐exposed children also significantly differed in medical assistance and race/ethnicity (all *P*'s ≤ 0.05). Among children aged 2–5, intervention children significantly differed from non‐respondent and non‐exposed children in age, BMI *z*‐score and weight category (all *P*'s ≤ 0.05). Additionally, children aged 2–5 in the intervention and non‐exposed groups significantly differed by sex, medical assistance and race/ethnicity. In contrast, among children aged 6–9, the intervention and non‐respondent groups significantly differed in age (*P* ≤ 0.01), and the intervention and non‐exposed group differed in medical assistance and race/ethnicity (all *P*'s ≤ 0.0001).

**Table 2a:** Characteristics of 2‐ to 9‐year‐old children at baseline in the FNPA risk assessment intervention group and comparison non‐respondent and non‐exposed groups

	Intervention (N = 2,724)		Non‐respondent (N = 3,324)		Non‐exposed (N = 4,599)		P‐value†	P‐value‡	
	Mean	SD	Mean	SD	Mean	SD			
Baseline age (years)	5.4	2.1	5.0	2.4	5.3	2.1	<0.0001	0.021	
BMI *z*‐score	0.46	1.12	0.36	1.17	0.29	1.14	0.0009	<0.0001	

	Category	N	%	N	%	N	%	P‐value†	P‐value‡
Sex	Female	1,351	49.6	1,622	48.8	2,208	48.0	0.536	0.189
	Male	1,373	50.4	1,702	51.2	2,391	52.0		
Weight category	Underweight	90	3.3	156	4.7	202	4.4	0.014	<0.0001
	Normal	1,810	66.5	2,242	67.5	3,271	71.1		
	Overweight	435	16.0	475	14.3	590	12.8		
	Obese	389	14.3	451	13.6	536	11.7		
Medical assistance	No	1,802	66.2	2,187	65.8	3,767	81.9	0.770	<0.0001
	Yes	922	33.9	1,137	34.2	832	18.1		
Race/ethnicity	Non‐Hispanic White	2,232	81.9	2,771	83.4	4,203	91.4	0.328	<0.0001
	Hispanic	279	10.2	319	9.6	120	2.6		
	Other	213	7.8	234	7.0	276	6.0		

BMI, body mass index; FNPA, family nutrition and physical activity; SD, standard deviation.

† Comparing intervention group versus non‐respondent group.

‡ Comparing intervention group versus non‐exposed group.

**Table 2b:** Characteristics of 2‐ to 5‐year‐old children at baseline in the FNPA risk assessment intervention group and comparison non‐respondent and non‐exposed groups

	Intervention (N = 1,617)		Non‐respondent (N = 2,133)		Non‐exposed (N = 2,746)		P‐value†	P‐value‡	
	Mean	SD	Mean	SD	Mean	SD			
Baseline age (years)	3.9	1.0	3.5	1.2	3.7	1.2	<0.0001	<0.0001	
BMI *z*‐score	0.44	1.14	0.26	1.19	0.17	1.17	<0.0001	<0.0001	

	Category	N	%	N	%	N	%	P‐value†	P‐value‡
Sex	Female	826	51.1	1,041	48.8	1,315	47.9	0.167	0.042
	Male	791	48.9	1,092	51.2	1,431	52.1		
Weight category	Underweight	56	3.5	121	5.7	157	5.7	0.0013	<0.0001
	Normal	1,096	67.8	1,485	69.6	2,007	73.1		
	Overweight	252	15.6	291	13.6	326	11.9		
	Obese	213	13.2	236	11.1	256	9.3		
Medical assistance	No	1,054	65.2	1,381	64.7	2,208	80.4	0.781	<0.0001
	Yes	563	34.8	752	35.3	538	19.6		
Race/ethnicity	Non‐Hispanic White	1,305	80.7	1,775	83.2	2,504	91.2	0.065	<0.0001
	Hispanic	179	11.1	222	10.4	71	2.6		
	Other	133	8.2	136	6.4	171	6.2		

BMI, body mass index; FNPA, family nutrition and physical activity; SD, standard deviation.

† Comparing intervention group versus non‐respondent group.

‡ Comparing intervention group versus non‐exposed group.

**Table 2c:** Characteristics of 6‐ to 9‐year‐old children at baseline in the FNPA risk assessment intervention group and comparison non‐respondent and non‐exposed groups

	Intervention (N = 1,107)		Non‐respondent (N = 1,191)		Non‐exposed (N = 1,853)		P‐value†	P‐value‡	
	Mean	SD	Mean	SD	Mean	SD			
Baseline age (years)	7.7	1.2	7.8	1.2	7.7	1.2	0.0006	0.363	
BMI *z*‐score	0.49	1.09	0.55	1.10	0.48	1.07	0.187	0.713	

	Category	N	%	N	%	N	%	P‐value†	P‐value‡
Sex	Female	525	47.4	581	48.2	893	48.1	0.515	0.686
	Male	582	52.6	610	51.8	960	51.9		
Weight category	Underweight	34	3.1	35	2.9	45	2.4	0.553	0.159
	Normal	714	64.5	757	63.6	1,264	68.2		
	Overweight	183	16.5	184	15.5	264	14.3		
	Obese	176	15.9	215	18.1	280	15.1		
Medical assistance	No	748	67.6	806	67.7	1,559	84.1	0.958	<0.0001
	Yes	359	32.4	385	32.3	294	15.9		
Race/ethnicity	Non‐Hispanic White	927	83.7	996	83.6	1,699	91.7	0.529	<0.0001
	Hispanic	100	9.0	97	8.1	49	2.6		
	Other	80	7.2	98	8.2	105	5.7		

BMI, body mass index; FNPA, family nutrition and physical activity; SD, standard deviation.

† Comparing intervention group versus non‐respondent group.

‡ Comparing intervention group versus non‐exposed group.

#### Feasibility

At baseline, parent response rate to FNPA risk assessment was 45% overall, 43% among parents of children aged 2–5 and 48% among parents of children aged 6–9. Data were collected in the clinic waiting room for 85% of the patients, whereas 15% completed the risk assessment in the patient portal.

#### Validation of risk score

The association between FNPA risk assessment summary scores and weight category at baseline was evaluated to validate the risk score. As shown in Table 3, the summary scores for intervention children significantly differed by weight category for children aged 2–9 and in both age groups (all *P*'s ≤ 0.01). The summary scores were grouped into tertiles by age and evaluated for odds of overweight or obesity at baseline 9, 11. In the present study, on average, the lowest tertile of summary scores was associated with greater odds ratio of being overweight (1.45, confidence interval [CI]: 1.22, 1.72), obese (1.70, CI: 1.36, 2.12) and severely obese (1.56, CI: 1.07, 2.29). Likewise, for children aged 2–5, the lowest tertile scores were associated with greater odds of being overweight (1.47, CI: 1.17, 1.85), obese (1.48, CI: 1.10, 1.99) and severely obese (1.34, CI: 0.84, 2.16). Similarly, for 6‐ to 9‐year‐olds, lower tertile scores were associated with greater odds ratio of being overweight (1.43, CI: 1.09, 1.87), obese (2.03, CI: 1.46, 2.83) and severely obese (2.09, CI: 1.09, 4.00).

**Table 3:** Association between baseline total FNPA risk assessment score with baseline weight status in the intervention group age 2–9 years (N = 2,724) and by age group 2–5 years (N = 1,617) and 6–9 years (N = 1,107)

	Underweight	Normal	Overweight	Obese	P‐value
Baseline FNPA, mean (SD)					
Age 2–9	*N* = 90	*N* = 1,810	*N* = 435	*N* = 389	<0.0001
	64.2 (6.3)	65.6 (5.9)	65.0 (5.8)	63.7 (6.0)	
Age 2–5	*N* = 56	*N* = 1,096	*N* = 252	*N* = 213	0.0033
	64.3 (4.9)	65.5 (5.8)	64.6 (5.7)	64.2 (5.6)	
Age 6–9	*N* = 34	*N* = 714	*N* = 183	*N* = 176	<0.0001
	64.1 (8.1)	65.9 (6.1)	65.5 (6.1)	63.0 (6.4)	

FNPA, family nutrition and physical activity; SD, standard deviation.

#### Baseline to 1‐year follow‐up

For children aged 2–9 (reported in parentheses) and aged 2–5 (reported in brackets), the observed effects followed the same pattern. The change in BMI *z*‐score from baseline to 1 year differed significantly by −0.05 (CI: −0.08, −0.02; *P* = 0.0013) [−0.09, CI: −0.13, −0.04; *P* = 0.0002] between the intervention and non‐respondent children, but no difference was observed between the intervention and non‐exposed children as shown in Table 4a (Table 4b). Children in the intervention group had a smaller increase in mean BMI *z*‐score (0.07 ± 0.63) [0.10 ± 0.71] than non‐respondent group (0.13 ± 0.63) [0.19 ± 0.71]. In a subsample of children with normal weight at baseline, the intervention versus non‐respondent group differed significantly in BMI *z*‐score change by −0.06 (CI: −0.10, −0.02; *P* = 0.0025) [−0.09, CI: −0.14, −0.03; *P* = 0.0013]. Children in the intervention group had a smaller increase in mean BMI *z*‐score (0.09 ± 0.62) [0.13 ± 0.68] than non‐respondent group (0.15 ± 0.61) [0.21 ± 0.67]. However, children with overweight at baseline in the intervention versus the non‐exposed group differed significantly in BMI *z*‐score change by 0.07 (CI: 0.02, 0.14; *P* = 0.016) [0.12, CI: 0.03, 0.22; *P* = 0.0096]. Children in the intervention group had an increase in mean BMI *z*‐score (0.02 ± 0.50) [0.02 ± 0.58], whereas those in the non‐exposed group decreased BMI *z*‐score (−0.06 ± 0.48) [−0.11 ± 0.56]. For children aged 6–9, there were no significant differences in BMI *z*‐score change at 1‐year follow‐up between intervention and non‐respondent or non‐exposed comparison groups (Table 4c). The BMI *z*‐score outcomes are reported from unadjusted models. In sensitivity analysis, adjusted models were evaluated (adjusting for baseline BMI *z*‐score, medical assistance, race and ethnicity) and found to be consistent with the reported unadjusted results (data not shown).

**Table 4a:** BMI z‐scores at baseline, 1‐year follow‐up and changes from baseline to 1‐year follow‐up compared between the intervention and non‐respondent or non‐exposed groups for subjects aged 2–9 years and by baseline weight category

Weight category	Timing	Intervention		Non‐respondent		Difference	P‐value
		N	Mean (SD)	N	Mean (SD)	Mean [95% CI]	
Age 2–9	Baseline	2,724	0.46 (1.12)	3,324	0.36 (1.17)	0.10 [0.04, 0.16]	0.0009
	1 year		0.54 (1.13)		0.49 (1.12)	0.05 [−0.01, 0.10]	0.117
	Change		0.07 (0.63)		0.13 (0.63)	−0.05 [−0.08, −0.02]	0.0013
Underweight	Baseline	90	−2.23 (0.70)	156	−2.17 (0.49)	−0.06 [−0.23, 0.10]	0.444
	1 year		−1.54 (0.99)		−1.31 (0.91)	−0.23 [−0.47, 0.01]	0.066
	Change		0.69 (1.20)		0.86 (0.94)	−0.16 [−0.46, 0.13]	0.232
Normal	Baseline	1,810	0.02 (0.64)	2,242	−0.04 (0.67)	0.05 [0.01, 0.09]	0.0093
	1 year		0.11 (0.81)		0.12 (0.79)	0.00 [−0.05, 0.04]	0.857
	Change		0.09 (0.62)		0.15 (0.61)	−0.06 [−0.10, −0.02]	0.0025
Overweight	Baseline	435	1.31 (0.18)	475	1.32 (0.17)	−0.01 [−0.04, 0.01]	0.264
	1 year		1.33 (0.53)		1.30 (0.58)	0.02 [−0.05, 0.10]	0.500
	Change		0.02 (0.50)		−0.02 (0.56)	0.04 [−0.03, 0.11]	0.282
Obese	Baseline	389	2.21 (0.52)	451	2.22 (0.49)	−0.01 [−0.08, 0.06]	0.721
	1 year		2.11 (0.64)		2.13 (0.58)	−0.01 [−0.10, 0.07]	0.765
	Change		−0.10 (0.54)		−0.10 (0.48)	0.00 [−0.07, 0.07]	0.994

Weight category	Timing	Intervention		Non‐exposed		Difference	P‐value
		N	Mean (SD)	N	Mean (SD)	Mean [95% CI]	
Age 2–9	Baseline	2,724	0.46 (1.12)	4,599	0.29 (1.14)	0.17 [0.12, 0.22]	<0.0001
	1 year		0.54 (1.13)		0.38 (1.14)	0.16 [0.10, 0.21]	<0.0001
	Change		0.07 (0.63)		0.09 (0.62)	−0.01 [−0.04, 0.02]	0.392
Underweight	Baseline	90	−2.23 (0.70)	202	−2.22 (0.51)	−0.02 [−0.16, 0.13]	0.847
	1 year		−1.54 (0.99)		−1.42 (0.92)	−0.12 [−0.36, 0.11]	0.298
	Change		0.69 (1.20)		0.80 (0.90)	−0.11 [−0.39, 0.17]	0.446
Normal	Baseline	1,810	0.02 (0.64)	3,271	−0.05 (0.67)	0.07 [0.03, 0.11]	0.0002
	1 year		0.11 (0.81)		0.04 (0.83)	0.07 [0.03, 0.12]	0.0029
	Change		0.09 (0.62)		0.09 (0.62)	0.00 [−0.04, 0.04]	0.990
Overweight	Baseline	435	1.31 (0.18)	590	1.32 (0.17)	−0.02 [−0.04, 0.01]	0.158
	1 year		1.33 (0.53)		1.27 (0.52)	0.06 [−0.01, 0.12]	0.076
	Change		0.02 (0.50)		−0.06 (0.48)	0.07 [0.02, 0.14]	0.016
Obese	Baseline	389	2.21 (0.52)	536	2.22 (0.49)	−0.01 [−0.08, 0.06]	0.752
	1 year		2.11 (0.64)		2.16 (0.59)	−0.04 [−0.12, 0.04]	0.305
	Change		−0.10 (0.54)		−0.07 (0.45)	−0.03 [−0.10, 0.03]	0.354

BMI, body mass index; SD, standard deviation.

**Table 4b:** BMI z‐scores at baseline, 1‐year follow‐up and changes from baseline to 1‐year follow‐up compared between the intervention and non‐respondent or non‐exposed groups for subjects aged 2–5 years and by baseline weight category

Weight category	Timing	Intervention		Non‐respondent		Difference	P‐value
		N	Mean (SD)	N	Mean (SD)	Mean [95% CI]	
Age 2–5	Baseline	1,617	0.44 (1.14)	2,133	0.26 (1.19)	0.18 [0.11, 0.26]	<0.0001
	1 year		0.54 (1.16)		0.45 (1.13)	0.10 [0.02, 0.17]	0.103
	Change		0.10 (0.71)		0.19 (0.71)	−0.09 [−0.13, −0.04]	0.0002
Underweight	Baseline	56	−2.26 (0.75)	121	−2.19 (0.49)	−0.07 [−0.29, 0.15]	0.539
	1 year		−1.47 (1.15)		−1.21 (0.93)	−0.25 [−0.57, 0.07]	0.120
	Change		0.79 (1.25)		0.98 (0.92)	−0.19 [−0.56, 0.19]	0.323
Normal	Baseline	1,096	0.02 (0.65)	1,485	−0.07 (0.68)	0.09 [0.04, 0.14]	0.0005
	1 year		0.15 (0.82)		0.14 (0.82)	0.01 [−0.06, 0.07]	0.858
	Change		0.13 (0.68)		0.21 (0.67)	−0.09 [−0.14, −0.03]	0.0013
Overweight	Baseline	252	1.30 (0.18)	291	1.31 (0.17)	−0.01 [−0.04, 0.02]	0.581
	1 year		1.32 (0.60)		1.28 (0.64)	0.03 [−0.07, 0.14]	0.526
	Change		0.02 (0.58)		−0.03 (0.63)	0.04 [−0.06, 0.15]	0.419
Obese	Baseline	213	2.31 (0.62)	236	2.30 (0.59)	0.00 [−0.11, 0.12]	0.930
	1 year		2.19 (0.78)		2.18 (0.72)	0.01 [−0.13, 0.15]	0.904
	Change		−0.12 (0.68)		−0.12 (0.61)	0.00 [−0.12, 0.12]	0.954

Weight category	Timing	Intervention		Non‐exposed		Difference	P‐value
		N	Mean (SD)	N	Mean (SD)	Mean [95% CI]	
Age 2–5	Baseline	1,617	0.44 (1.14)	2,746	0.17 (1.17)	0.27 [0.20, 0.35]	<0.0001
	1 year		0.54 (1.16)		0.31 (1.14)	0.24 [0.17, 0.31]	<0.0001
	Change		0.10 (0.71)		0.14 (0.70)	−0.04 [−0.08, 0.00]	0.081
Underweight	Baseline	56	−2.26 (0.75)	157	−2.23 (0.51)	−0.03 [−0.24, 0.19]	0.810
	1 year		−1.47 (1.15)		−1.29 (0.96)	−0.17 [−0.51, 0.17]	0.271
	Change		0.79 (1.25)		0.94 (0.92)	−0.15 [−0.51, 0.22]	0.350
Normal	Baseline	1,096	0.02 (0.65)	2,007	−0.10 (0.68)	0.12 [0.08, 0.17]	<0.0001
	1 year		0.15 (0.82)		0.04 (0.84)	0.10 [0.04, 0.16]	0.0010
	Change		0.13 (0.68)		0.15 (0.67)	−0.02 [−0.07, 0.03]	0.421
Overweight	Baseline	252	1.30 (0.18)	326	1.31 (0.17)	−0.01 [−0.03, 0.02]	0.713
	1 year		1.32 (0.60)		1.20 (0.61)	0.12 [0.02, 0.22]	0.020
	Change		0.02 (0.58)		−0.11 (0.56)	0.12 [0.03, 0.22]	0.0096
Obese	Baseline	213	2.31 (0.62)	256	2.32 (0.61)	−0.01 [−0.12, 0.10]	0.877
	1 year		2.19 (0.78)		2.21 (0.77)	−0.02 [−0.16, 0.12]	0.738
	Change		−0.12 (0.68)		−0.10 (0.61)	−0.02 [−0.13, 0.10]	0.799

BMI, body mass index; SD, standard deviation.

**Table 4c:** BMI z‐scores at baseline, 1‐year follow‐up and changes from baseline to 1‐year follow‐up compared between the intervention and non‐respondent or non‐exposed groups for subjects aged 6–9 years and by baseline weight category

Weight category	Timing	Intervention		Non‐respondent		Difference	P‐value
		N	Mean (SD)	N	Mean (SD)	Mean [95% CI]	
Age 6–9	Baseline	1,107	0.49 (1.09)	1,191	0.55 (1.10)	−0.06 [−0.15, 0.03]	0.187
	1 year		0.53 (1.10)		0.57 (1.10)	−0.04 [−0.13, 0.05]	0.343
	Change		0.04 (0.49)		0.02 (0.43)	0.02 [−0.02, 0.05]	0.382
Underweight	Baseline	34	−2.19 (0.63)	35	−2.10 (0.47)	−0.10 [−0.36, 0.17]	0.468
	1 year		−1.67 (0.64)		−1.67 (0.72)	0.00 [−0.33, 0.32]	0.979
	Change		0.52 (1.11)		0.43 (0.88)	0.09 [−0.39, 0.57]	0.700
Normal	Baseline	714	0.01 (0.64)	757	0.03 (0.64)	−0.02 [−0.09, 0.05]	0.541
	1 year		0.05 (0.78)		0.07 (0.72)	−0.01 [−0.09, 0.07]	0.809
	Change		0.04 (0.50)		0.03 (0.43)	0.01 [−0.04, 0.06]	0.653
Overweight	Baseline	183	1.31 (0.18)	184	1.34 (0.18)	−0.02 [−0.06, 0.02]	0.246
	1 year		1.33 (0.40)		1.32 (0.47)	0.01 [−0.08, 0.10]	0.847
	Change		0.02 (0.37)		−0.01 (0.42)	0.03 [−0.05, 0.11]	0.459
Obese	Baseline	176	2.09 (0.33)	215	2.14 (0.32)	−0.04 [−0.11, 0.02]	0.204
	1 year		2.03 (0.40)		2.07 (0.37)	−0.04 [−0.12, 0.03]	0.260
	Change		−0.07 (0.29)		−0.07 (0.24)	0.00 [−0.05, 0.05]	0.945

Weight category	Timing	Intervention		Non‐exposed		Difference	P‐value
		N	Mean (SD)	N	Mean (SD)	Mean [95% CI]	
Age 6–9	Baseline	1,107	0.49 (1.09)	1,853	0.48 (1.07)	0.02 [−0.07, 0.10]	0.713
	1 year		0.53 (1.10)		0.49 (1.13)	0.04 [−0.04, 0.12]	0.338
	Change		0.04 (0.49)		0.01 (0.46)	0.03 [−0.01, 0.06]	0.160
Underweight	Baseline	34	−2.19 (0.63)	45	−2.17 (0.50)	−0.03 [−0.29, 0.24]	0.838
	1 year		−1.67 (0.64)		−1.86 (0.56)	0.19 [−0.08, 0.47]	0.157
	Change		0.52 (1.11)		0.30 (0.64)	0.22 [−0.21, 0.65]	0.272
Normal	Baseline	714	0.01 (0.64)	1,264	0.02 (0.65)	−0.01 [−0.07, 0.05]	0.762
	1 year		0.05 (0.78)		0.03 (0.82)	0.02 [−0.05, 0.10]	0.520
	Change		0.04 (0.50)		0.01 (0.51)	0.03 [−0.01, 0.08]	0.160
Overweight	Baseline	183	1.31 (0.18)	264	1.34 (0.17)	−0.03 [−0.06, 0.01]	0.106
	1 year		1.33 (0.40)		1.35 (0.37)	−0.01 [−0.09, 0.06]	0.699
	Change		0.02 (0.37)		0.01 (0.33)	0.01 [−0.05, 0.08]	0.698
Obese	Baseline	176	2.09 (0.33)	280	2.14 (0.33)	−0.04 [−0.10, 0.02]	0.205
	1 year		2.03 (0.40)		2.11 (0.35)	−0.08 [−0.15, −0.01]	0.031
	Change		−0.07 (0.29)		−0.03 (0.22)	−0.04 [−0.09, 0.01]	0.125

BMI, body mass index; SD, standard deviation.

#### Post hoc clustering analyses

In multilevel modelling (data not shown), the percent of variation explained by clinic site and provider was <1% in the overall model, suggesting that clinic and provider characteristics accounted for a minimal amount of change in BMI *z*‐score (similar results were observed when stratifying within intervention, non‐respondent and non‐exposed participants). To confirm this assumption, multilevel models that account for clinic and provider clustering, clinic characteristics and a provider characteristic resulted in parameter estimates of a similar magnitude and significance level to those derived from simple analyses.

## Discussion

This is the first study to demonstrate the feasibility and clinical effectiveness of implementing an automated risk assessment during WCV for paediatric preventive care. Lower FNPA summary scores were associated with greater odds for overweight, obesity and severe obesity among school‐aged children, consistent with prior research, as well as among preschool‐aged children, a population previously uninvestigated 9, 11. On average, children whose parent completed of the FNPA risk assessment, and thus had exposure to the intervention, experienced smaller increases in BMI *z*‐score compared with non‐respondent children. Results were similar for children with a normal weight at baseline and those aged 2–5 but not for children aged 6–9. However, children (aged 2–9 and 2–5) with overweight at baseline had a larger increase in BMI *z*‐score compared with the non‐exposed group. These results suggest that exposure to the risk assessment may promote healthier growth patterns for children aged 2–9 and 2–5, specifically for children with normal weight at baseline. It is not clear why children with overweight exposed to the intervention had unhealthier growth patterns and why significant effects were not observed among children aged 6–9. Results could be due to the intervention emphasizing behavioural and environmental factors within the family and at home, a setting where preschool‐aged children spend more time as compared with school‐aged children 18.

Poor nutrition, inadequate physical activity, excessive screen time and sedentary activity, and poor sleep habits are risk factors for childhood obesity; however, clinicians need a reliable mechanism to assess risk, assurance that the score is evidence based, and skills for translating the risk score into preventive counselling. This study demonstrates feasibility and sustainability of this approach. In the present health system, FNPA risk assessment completion rates, 5 years after introduction, exceed 50% confirming the sustainability of automated mechanism to prompt data collection, but additional efforts are needed to improve completion rates. In comparison with paper‐version or interview‐administered tools that require human resources, automation is feasible, sustainable and aided with multiple collection opportunities (e.g. parent portal and clinic staff encouragement) 19. Implementing an automated mechanism to collect and integrate risk assessment data for use in routine WCV into providers' workflow is feasible, but clinician utilization of the risk score and counselling practices should be continuously evaluated.

The FNPA risk assessment is a valid tool to identify risk factors associated with overweight and obesity among school‐aged and preschool‐aged children. The results with children aged 6–9 align with previous research demonstrating that the FNPA summary score is associated with odds of overweight, obesity and severe obesity 9, 11, 14. Prevention prior to age 5 years is essential as obesity tends to persist once established 20, 21; however, no risk assessments were identified for children as young as age 2 years when the study was designed. Recently, the Healthy Kids obesity risk assessment has shown face, content, convergent and predictive validity in preschool‐aged children with lower household income 22. Both FNPA and Healthy Kids risk assessments offer potential value to clinicians to involve parents in self‐assessing risk using validated tools. This study did not evaluate parent experience or behaviour change following FNPA risk assessment; however, promising results have been observed in earlier studies 15, 16.

Enhancing BMI screening with FNPA risk assessment at routine WCVs was beneficial for children aged 2–9 and 2–5 overall, specifically those with normal weight at baseline, compared with children whose parents did not complete FNPA at baseline. Change in BMI *z*‐score between baseline and 1‐year follow‐up favoured the intervention indicating healthier growth patterns. The overall difference in favour of the intervention group was in the same direction and equivalent to the effect of school‐based preventive interventions using more intense approaches and rigorous study designs 23. This study addresses the lack of preventive interventions in primary care with promising results 23. Albeit, these findings may be confounded by survey response bias. Because of lack of randomization, we cannot rule out that parents of children who completed the FNPA assessment were more motivated to address obesity risk factors than those who failed to respond.

Overall, no significant differences were observed when comparing the intervention and non‐exposed groups. However, change in BMI *z*‐score among children with overweight favoured the non‐exposed group rather than the intervention group. Preventive counselling related to healthy eating and physical activity could have occurred in non‐intervention clinics to benefit the non‐exposed group but was unlikely 24, 25, 26. Alternatively, provider‐level factors such as years of practice or duration of the relationship with the family may offer insight for future research.

Inconsistent findings between the intervention and comparison groups may be attributed to differences in socio‐economic status or race and ethnicity as prior research indicates strong influences from these social determinants on BMI 27. Regardless, comparisons between the intervention versus non‐respondent groups are more ecologically valid as these individuals were offered the same opportunity and received care from the same providers in the same clinics. Importantly, these comparisons underscore the value of the automated risk assessment and clinical decision support tools over provider training alone 28, 29. Without access to the risk assessment data, summary score, talking points and educational materials, provider training offered no observed protective benefit on BMI.

The null findings observed among children aged 6–9 reinforce the guidance from the US Preventive Services Task Force 3. In this older group, BMI screening plus risk assessment should inform preventive counselling and the provision of or referral to proven, evidence‐based interventions. One occurrence of a WCV with BMI screening, risk assessment and preventive counselling may not be enough to prevent or reduce obesity. Future studies should utilize longitudinal designs to evaluate whether regular, sequential WCVs with BMI screening and risk assessment are associated with prevention and remission of obesity, consistent with natural observations 30.

Strengths of the study include the large sample size and the quasi‐experimental design that compared outcomes under ecologically sound, naturalized conditions. The gains in external validity in this case outweigh the loss of internal validity associated with the lack of a truly experimental design. However, these findings should be considered in context of observed limitations. Implementation feasibility was evaluated by one factor, but clinic staff, patient experience, provider acceptance and years of practice should be evaluated. The relationship between FNPA risk score and baseline weight did not control for parent BMI, a covariate that influenced this relationship in prior studies 9, 11. Significant differences between the groups on nearly all characteristics and inconsistent results between the intervention and comparison groups could be explained by lack of random assignment at the clinic or individual level and uncollected variables.

## Conclusion

The present study is the first to demonstrate the feasibility of an automated and validated risk assessment in routine paediatric well‐child care for preschool‐aged and school‐aged children. Integrating routine BMI screening with parental assessment of family practices, child behaviours and home environmental risk factors as clinical decision support in the child's EHR resulted in favourable weight outcomes, particularly among preschool‐aged children, when compared with children who received care in the same clinics but who did not have a completed risk assessment. This approach is feasible, useful and sustainable and can be applied to improve childhood obesity prevention efforts in clinical settings.

## Funding

Geisinger Clinical Innovations and Geisinger Obesity Institute provided support for the project.

## Conflict of Interest Statement

The authors declared no conflicts of interest.
